# Antioxidative Responses to Pre-Storage Hot Water Treatment of Red Sweet Pepper (*Capsicum annuum* L.) Fruit during Cold Storage

**DOI:** 10.3390/foods10123031

**Published:** 2021-12-06

**Authors:** Jirarat Kantakhoo, Yoshihiro Imahori

**Affiliations:** Laboratory of Postharvest Physiology, Graduate School of Life and Environmental Sciences, Osaka Prefecture University, 1-1 Gakuen-cho, Nakaku, Sakai, Osaka 599-8531, Japan; jirarattim@gmail.com

**Keywords:** *Capsicum annuum* L., hot water treatment, chilling injury, ascorbate-glutathione cycle

## Abstract

The effects of hot water treatments on antioxidant responses in red sweet pepper (*Capsicum annuum* L.) fruit during cold storage were investigated. Red sweet pepper fruits were treated with hot water at 55 °C for 1 (HWT-1 min), 3 (HWT-3 min), and 5 min (HWT-5 min) and stored at 10 °C for 4 weeks. The results indicated that HWT-1 min fruit showed less development of chilling injury (CI), electrolyte leakage, and weight loss. Excessive hot water treatment (3 and 5 min) caused cellular damage. Moreover, HWT-1 min slowed the production of hydrogen peroxide and malondialdehyde and promoted the ascorbate and glutathione contents for the duration of cold storage as compared to HWT-3 min, HWT-5 min, and control. HWT-1 min enhanced the ascorbate-glutathione cycle associated with ascorbate peroxidase, monodehydroascorbate reductase, dehydroascorbate reductase, and glutathione reductase, but it was less effective in simulating catalase activity. Thus, HWT-1 min could induce CI tolerance in red sweet pepper fruit by activating the ascorbate-glutathione cycle via the increased activity of related enzymes and the enhanced antioxidant level.

## 1. Introduction

Red sweet pepper (*Capsicum annuum* L.) is an important crop of the dominant horticultural products in terms of the global economy. It is grown worldwide in tropics and subtropics. More than 65% of the production area is in Asia; China is the largest producer, followed by Mexico, Turkey, Indonesia, Spain, and the USA [1]. Red sweet pepper fruit contains nutrition value (a good source of vitamin A and C) and bioactive materials, such as phenolic compounds and carotenoids [2,3]. However, red sweet pepper fruit are a perishable commodity due to mechanical damage and microbial deterioration related to softening along with fruit ripening when stored at ambient temperature, resulting in a short shelf-life after harvest. Thus, a suitable storage method is necessary to maintain the quality of red sweet pepper fruit.

Low-temperature storage maintains the quality of horticultural products, and is required to extend the storage duration for horticultural products, particularly for long-distance transport or export quarantine treatments that take 21–24 days before marketing [4,5]. The shelf life of peppers in storage is limited by decay, loss of water during storage, and sensitivity to cold stress. It is reported that the optimum storage temperature for peppers is indicated to be 12 °C [6]. However, at storage temperatures below the optimum temperature, peppers often occur chilling injury (CI) according to their chilling sensitivity. CI symptoms indicates surface pitting, calyx discoloration, water-soaked areas, and shriveling related with moisture loss [7]. The development of CI symptoms relates to several factors. The severity level of CI is subject to the storage time and storage temperature interaction. Generally, after exposure to lower chilling temperatures and longer duration, horticultural products suffer more CI [8].

Several stress in plant is caused by change in the environment conditions [9]. Environmental stress excessively induces reactive oxygen species (ROS) production and provokes oxidative stress to the plant. Oxidative stress is exhibited when the ROS produces over the capacity that maintains homeostasis in plant cellular redox. Imbalances between the generation and detoxification for ROS are CI responses in horticultural crops [10,11]. Lipid peroxidation in a plant is the first evidence of the illustration of CI. Malondialdehyde (MDA) is generated by the non-enzymatic oxidation and enzymatic destruction of polyunsaturated fatty acids in plant cell membranes. The level of MDA is usually a marker of plant oxidative stress, and its level is used to evaluate lipid peroxidation [9,12]. Plants react to oxidative stress by non-enzymatic and enzymatic systems that sustain the equilibrium between ROS generating and scavenging. This system includes both antioxidants of ascorbate (AsA) and glutathione (GSH), and scavenging enzymes that consist of catalase (CAT) and ascorbate-glutathione (AsA-GSH) cycle-related enzymes composed of ascorbate peroxidase (APX), monodehydroascorbate reductase (MDHAR), dehydroascorbate reductase (DHAR), and glutathione reductase (GR). The AsA-GSH cycle, which implicates antioxidants and antioxidative enzymes, improves the chilling resistance of plants [13,14].

Hot water treatment can be utilized to inhibit fruit ripening or to increase CI tolerance. The alleviative effect of CI by hot water treatment has been revealed in many horticultural products [15], such as cucumber [16], zucchini [17], mume [18], banana [19], orange [20], and many other crops [21,22,23]. The mechanism to mitigate CI in fresh fruits and vegetables by postharvest heat treatment could be attributed to (1) improving the membrane integrity by the increase of unsaturated fatty acids and saturated fatty acids ratio; (2) enhancing heat shock protein gene expression and accumulation; (3) enhancing the antioxidant system; (4) enhancing the arginine pathways, which result in the accumulation of signaling molecules that play critical roles in improving chilling tolerance; (5) altering phenylalanine ammonialyase and polyphenol oxidase enzyme activities; and (6) enhancing sugar metabolism [21]. 

Hot water immersion is a nonchemical and safe postharvest treatment [13]. Pre-storage with moderate heat treatment of fresh commodities not only improves their heat stress resistance but also promotes tolerance to other stresses [22]. In scanning electron microscopy analysis of hot water-treated sweet pepper fruit, it was indicated that hot water immersion markedly firmed the surface and that cracks in the epidermis were sealed due to recrystallization or melting of the wax layer, thus maintaining the fruit quality during cold storage [24]. Exposing to sub-lethal temperatures induces thermos-tolerance, which can protect horticultural products from subsequent introduction into lethal temperatures [15]. However, the responses of commodities to hot water treatments differ depending on the treatment temperature and time of exposure to hot water. It is important that the suitable conditions of hot water immersion are established for low-temperature storage. 

Thus, the object in our study was to analyze the influences of different immersion durations (1, 3, and 5 min) of hot water immersion at 55 °C on antioxidant responses by monitoring the oxidative stress level and the AsA-GSH cycle in red sweet pepper fruit during low-temperature storage at 10 °C, and to determine the suitable conditions of hot water treatment.

## 2. Materials and Methods

### 2.1. Plant Materials and Treatments

Red sweet pepper (*Capsicum annuum* L.) cv. Habataki fruits were obtained at the red ripening stage from an agricultural field in Kochi Prefecture, Japan. Fruits of uniform size that were free of defects and had been delivered immediately to the laboratory were selected for this study. In total, 144 fruits of sweet pepper fruits were cleaned with water, air-dried at 20 °C for 15 min, and divided into four groups (36 fruits each). According to a preliminary experiment based on cold storage in fruit treated by hot water at different temperatures, suitable temperatures for hot water treatment were selected. The results showed that hot water treatment at 55 °C had better effects on alleviating CI. One group (control) was kept without being treated. The others were immersed in hot water at 55 °C for 1 (HWT-1 min), 3 (HWT-3 min), and 5 min (HWT-5 min), respectively, and then quick cooled until room temperature. After air drying, 3–4 fruits each were packed in a polyethylene film bag (38 cm × 26 cm with 0.03 mm thickness and four 5 mm holes on each side). In all treatments, three bags of fruits represented three replications. The pepper fruits were stored in a laboratory refrigerator at 10 °C and relative humidity (RH) of 95% for 4 weeks. Samples were taken every 7 days during storage to measure the quality attributes (weight loss; L*, a*, and b*; and overall quality); CI index; and CI incidence; and to determine electrolyte leakage. Fruit pericarp tissues were cut into roughly 1 cm pieces, immediately immersed in liquid nitrogen, and then preserved at −80 °C until further analysis.

### 2.2. Chilling Injury

The chilling injury symptom was evaluated on the level of surface pitting in accordance with the methods described by Wang et al. [25]. CI was measured according to a scale of 6 levels; 0, no pitting; 1, trace (0% < injury ≤ 10%); 2, slight (10% < injury ≤ 20%); 3, regular (20% < injury ≤ 30%); 4, moderate (30% < injury ≤ 50%); 5, severe (injury ≥ 50%). CI index and CI incidence were determined by the following formula: CI index = (sum (CI scale × number of fruit at the CI scale))/total number of evaluated fruit; CI incidence (%) = (the number of CI fruit/the total number of evaluated fruits) × 100.

### 2.3. Quality Attributes 

Red sweet peppers were weighed at the beginning and the end of each storage period. The variance between the two weigh values was calculated to be the total weight reduction and was indicated as a percent on a fresh weight basis.

Fruit color was indicated by measuring L* (lightness), a* (redness), and b* (yellowness) using a color difference meter (Nippon Denshoku, ZE 6000, Tokyo, Japan). Each fruit was measured with three replications.

The overall quality of the fruit was assessed by the method of Özden and Bayindirli [6]. The quality was evaluated subjectively for their flavor and appearance according to the following scale: 5 = excellent, 3 = marketable level, and 1 = unusable.

### 2.4. Electrolyte Leakage

Electrolyte leakage was determined according to the method of Endo et al. [13]. Twenty disks (10 mm × 2 mm) of fruit pericarp tissue from each fruit were cut using a cork borer and then rinsed with distilled water before immersion in 30 mL of double-distilled water in glass vials. After incubation for 2 h at 25 °C, the initial electrolyte conductivity was evaluated with a digital electrolyte conductivity meter (DKK-TOA, MM-41DP, Japan). The disks were boiled for 30 min to complete the electrolyte leakage, and the final electrolyte conductivity was determined. The percent of electrolyte leakage was expressed as a relative between the initial and final electrolyte conductivity.

### 2.5. Malondialdehyde Content

The level of lipid peroxidation was expressed by the MDA level based on the thiobarbituric acid (TBA) reaction following the method of Dipierro and De Leonardis [26]. Two grams of frozen sample were extracted in 10 mL of cold 0.1% trichloroacetic acid (TCA). The extract was passed through filter paper and centrifuged at 20,000× *g* for 10 min at 4 °C. Then, 1 mL supernatant of extract was mixed with 4 mL of 0.5% TBA in 20% TCA and then was heated at 95 °C for 30 min and then quick cooled to room temperature. The absorbance was determined spectrophotometrically at 532 and 600 nm (Jasco V-530, Jasco, Tokyo, Japan), after being centrifuged at 20,000× *g* for 10 min at 4 °C. The MDA content was calculated using the extinction coefficient, 155 mM^−1^cm^−1^. 

### 2.6. Hydrogen Peroxide Content

The content of hydrogen peroxide (H_2_O_2_) was determined by the chromogenic peroxidase-coupling method following the procedures of Veljovic-Jovanovic et al. [27]. Three grams of frozen sample were extracted in 12 mL of cold 1 M HClO_4_, then passed through filter paper. After centrifugation at 12,000× *g* for 10 min at 4 °C, the supernatant of extract was neutralized to pH 5.6 by 5 M K_2_CO_3_ and then centrifuged at 12,000× *g* for 10 min at 4 °C to eliminate insoluble KClO_4_. To oxidize ascorbate before incubation, the supernatant was incubated with 1-unit ascorbate oxidase for 10 min. Then, 1 mL of neutralized supernatant was reacted with the reaction mixture solution of 0.1 M phosphate buffer (pH 6.5) containing 16.5 mM 3-(dimethylamino) benzoic acid, 0.35 mM 3-methyl-2-benzothiazoline hydrazine, and 250 ng horseradish peroxidase. The absorbance was measured spectrophotometrically at 590 nm (Jasco V-530, Jasco, Tokyo, Japan) and monitored at 25 °C. The content of hydrogen peroxide was determined from a 25–100 µM H_2_O_2_ standard curve.

### 2.7. Antioxidant Content 

#### 2.7.1. Ascorbate and Dehydroascorbate 

The contents of ascorbate and dehydroascorbate (DHA) were determined following the procedures of Stevens et al. [28]. Three grams of frozen sample were homogenized in 12 mL of cold 6% TCA. The extract was passed through two layers of Miracloth (Calbiochem, San Diego, CA, USA). Two assays were performed on each sample to analyze the total ascorbate and reduced ascorbate. The first assay measured the total ascorbate (incubation with 5 mM dithiothreitol (DTT) for reduction of the oxidized ascorbate), and another assay measured the reduced ascorbate (without DTT). The content of DHA was determined from the difference between the total and the reduced ascorbate content. In each sample, after the filtrate (1 mL) was mixed with 1 mL of 0.4 M phosphate buffer (pH 7.4) with 5 mM DTT or 0.4 M phosphate buffer (pH 7.4), it was incubated at 37 °C for 20 min. After incubation, the reaction solution was added with 5 mL of 0.5% *N*-ethyl malemide for the total ascorbate assay, or 5 mL of 0.4 M phosphate buffer (pH 7.4) for the reduced ascorbate assay, and left for 1 min at room temperature, and finally was added with 4 mL of color reagent. The absorbance was determined spectrophotometrically at 550 nm (Jasco V-530, Jasco, Tokyo, Japan) after 40 min incubation at 37 °C. The AsA content was calculated from a standard curve. The color reagent was as follows: solution A comprised of 31% orthophosphoric acid, 4.6% TCA, and 0.6% iron chloride; and solution B, which included 4% 2,2-dipyridyl (made up of 70% ethanol). Solutions A and B were mixed 2.75 parts (A) to 1 part (B). The content of AsA was estimated by a standard curve.

#### 2.7.2. Reduced Glutathione and Oxidized Glutathione 

The contents of GSH and GSSG contents were determined using the 5,5′-dithiobis-(2-nitrobenzoic acid) (DTNB) and glutathione reductase procedure as detailed by Griffith [29], with certain modifications. Frozen fruit tissue (5 g) was homogenized with 10 mL of cold 5% sulphosalicylic acid and passed through two layers of Miracloth (Calbiochem, San Diego, CA, USA). The homogenate was centrifuged at 12,000× *g* for 10 min at 4 °C. The extract was neutralized to pH 7.0 using with 7.5 M triethanolamine. Each neutralized solution was divided into 2 assays. One (1 mL) was used to determine the total glutathione content (GSH and GSSG). Another (1 mL) was reacted with 20 µL of 2-vinylpyridine for 60 min at 20 °C to allow the derivatization of GSH and the detection of only GSSG in the subsequent assay. The assay was carried out by adding 50 µL of the sample, 150 µL of 125 mM sodium phosphate buffer (pH 6.5) including 6.3 mM EDTA, 700 µL of 0.3 mM NADPH, 100 µL of 0.6 mM DTNB, and 10 µL of 50 units mL^−1^ GR in a cuvette with a 1 cm light path. The absorbance was monitored spectrophotometrically at 412 nm (Shimadzu UV-1800, Shimadzu, Japan) for 120 s at a temperature of 30 °C. Total glutathione and GSSG contents were estimated from the standard curve of GSH using 25–100 µL.

### 2.8. Antioxidant Enzyme Extraction and Assay

Enzyme extraction was performed following the method of Ishikawa et al. [30], with certain modifications. Frozen fruit tissue (5 g) was homogenized in a cooled mortar and pestle with 20 mL of cold 50 mM potassium phosphate buffer (pH 7.0) containing 20% sorbitol, 1 mM ascorbate, 1 mM EDTA, and 4% polyvinylpyrrolidone. The homogenate was passed through two layers of Miracloth (Calbiochem, San Diego, CA, USA) and centrifuged at 15,000× *g* for 15 min at 4 °C. 

The activity of CAT was determined according to the procedure established by Aebi [31]. H_2_O_2_ consumption was recorded as the decrease in absorbance at 240 nm (Shimadzu UV-1800, Shimadzu, Japan) at 25 °C for 120 s. The calculation of CAT activity used an extinction coefficient of 39.4 mM^−1^ cm^−1^. 

The activity of APX was estimated according to the procedure of Nakano and Asada [32]. The ascorbate oxidation was recorded as the absorbance decrease at 290 nm (Shimadzu UV-1800, Shimadzu, Japan) at 25 °C for 120 s. The calculation of APX activity used an extinction coefficient of 2.8 mM^−1^ cm^−1^.

The activity of MDHAR was estimated according to the procedure of Hossain and Asada [33]. The NADH oxidation was recorded as the absorbance decrease at 340 nm (Shimadzu UV-1800, Shimadzu, Japan) at 25 °C for 120 s. The calculation of MDHAR activity used an extinction coefficient of 6.2 mM^−1^ cm^−1^.

The activity of DHAR was estimated according to the procedure of Hossain and Asada [33]. The generation of ascorbate was recorded as the increase in absorbance at 265 nm (Shimadzu UV-1800, Shimadzu, Japan) at 25 °C for 120 s. The calculation of DHAR activity used an extinction coefficient of 14 mM^−1^ cm^−1^.

The activity of GR was estimated according to the procedure of Klapheck et al. [34]. The NADPH oxidation was recorded as the absorbance decrease at 340 nm (Shimadzu UV-1800, Shimadzu, Japan) at 25 °C for 120 s. The calculation of GR activity used an extinction coefficient of 6.2 mM^−1^ cm^−1^.

### 2.9. Determination of Protein 

The protein content in extracts was measured by the method of Bradford [35], using bovine serum albumin as the standard.

### 2.10. Data Analysis

The data were subjected to two-way analysis of variance (treatments and storage weeks) for a completely randomized design using SPSS (SPSS 16.0 for Windows) statistical software. Mean comparisons were determined by Duncan’s multiple range test (DMRT) at a significance level of 0.05 (*p* < 0.05). The data were reported as the means ± S.E. (standard error).

## 3. Results

### 3.1. Changes in the Chilling Injury Index and Chilling Injury Incidence

**Figure 1:**
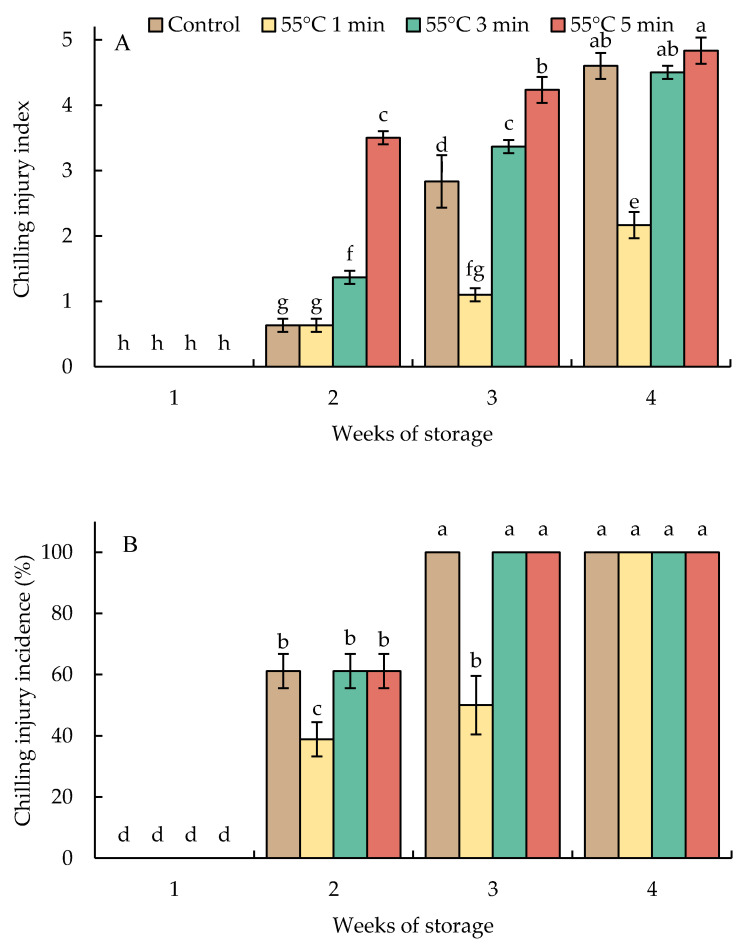
Effect of hot water treatment (55 °C for 1, 3, 5 min) on (**A**) chilling injury index and (**B**) chilling injury incidence in red sweet pepper fruit during storage at 10 °C. Data represent means ± S.E. (n = 3). Different letters on bars are significantly different (*p* < 0.05).

**Figure 2:**
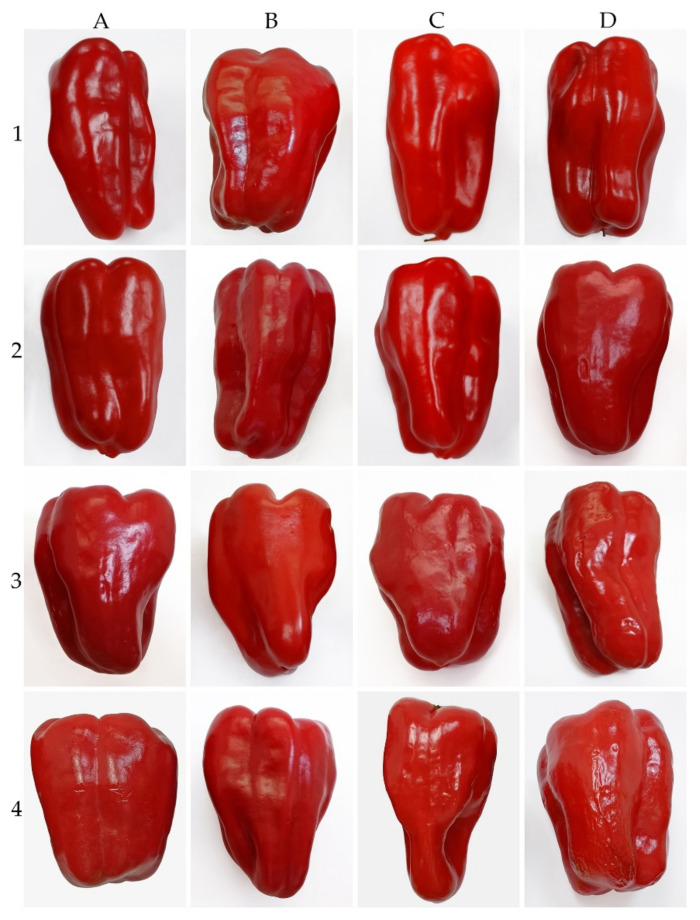
Effect of hot water treatment at 55 °C for (**A**) 0 min (control), (**B**) 1 min, (**C**) 3 min, and (**D**) 5 min on virtual symptoms of CI in red sweet pepper fruit during storage at 10 °C for 4 weeks (1, 2, 3, 4).

Hot water-treated fruit and untreated fruit (control) presented CI symptoms and incidence by week 2 and developed continuously with storage time (Figure 1 and Figure 2). Fruit heated at 55 °C for 1 min (HWT-1 min) exhibited a delay in the development of CI symptoms and a lower percentage of incidence than HWT-3 min, HWT-5 min, and the control fruit. By 2 weeks of storage, the CI incidence of 60% was shown in HWT-3 min, HWT-5 min, and the control fruit, and it reached 100% in week 3. In week 4, the severity of CI in HWT-1 min fruit was slight (CI index = 2), but damage was moderate to severe (CI index = 4.5–4.8) in control, HWT-3 min, and HWT-5 min fruit.

### 3.2. Changes in Quality Attributes

**Figure 3:**
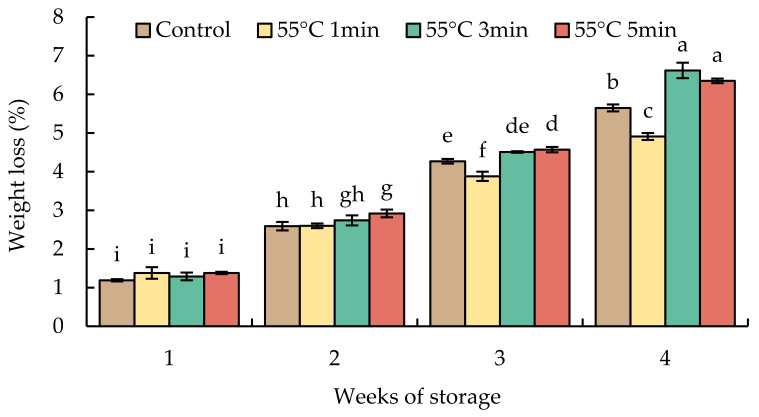
Effect of hot water treatment (55 °C for 1, 3, 5 min) on weight loss in red sweet pepper fruit during storage at 10 °C. Data represent means ± S.E. (n = 3). Different letters on bars are significantly different (*p* < 0.05).

Weight loss continued to increase over the storage period (Figure 3). The accumulated losses did not differ statistically after storage for 1 and 2 weeks. The HWT-3 min and HWT-5 min fruits exhibited significant loss of water by week 4 and had higher losses than HWT-1 min and control fruits. 

It was found that there was no significant difference of L*, a*, or b* between hot water-treated fruits and the control fruit during storage at 10 °C for 4 weeks (*p* > 0.05) (Figure S1).

**Figure 4:**
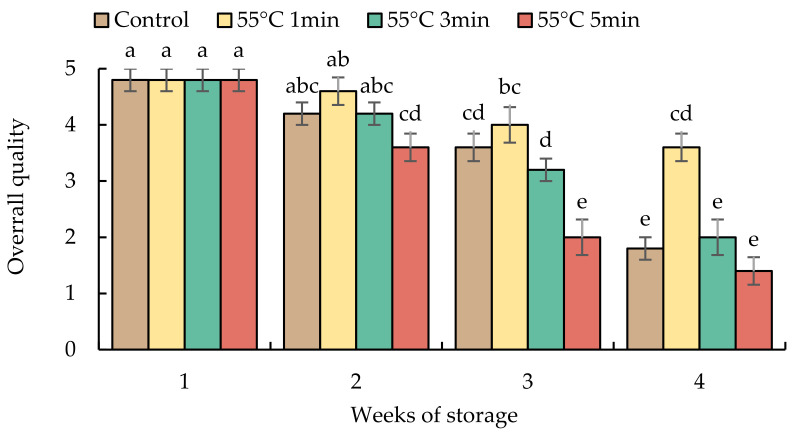
Effect of hot water treatment (55 °C for 1, 3, 5 min) on overall quality in red sweet pepper fruit during storage at 10 °C. Data represent means ± S.E. (n = 3). Different letters on bars are significantly different (*p* < 0.05).

The overall quality of sweet pepper fruits declined during prolonged storage in all treatments (Figure 4). HWT-1 min fruits had a higher overall quality score until the end of storage, while HWT- 5 min fruits lost the market potential after storage for 3 weeks; control and HWT-3 min fruits were below the marketable level at week 4 (as the score was less than 3).

### 3.3. Changes in Electrolyte Leakage

**Figure 5:**
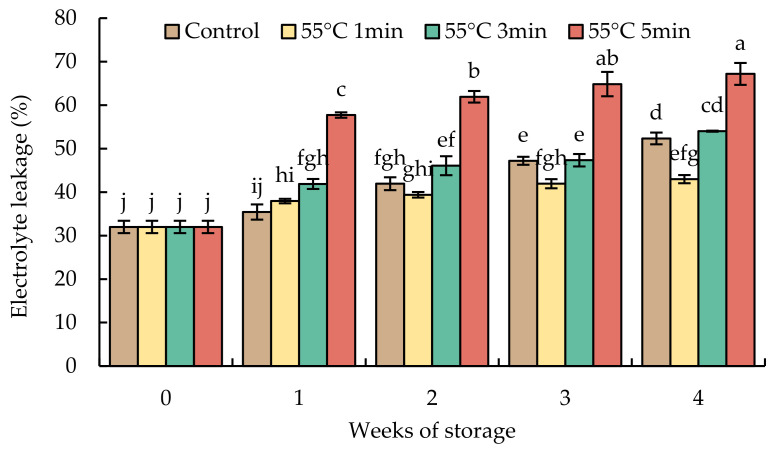
Effect of hot water treatment (55 °C for 1, 3, 5 min) on electrolyte leakage in red sweet pepper fruit during storage at 10 °C. Data represent means ± S.E. (n = 3). Different letters on bars are significantly different (*p* < 0.05).

The value of electrolyte leakage in the initial time was about 30% (Figure 5). After 4 weeks of storage, the electrolyte leakage sharply increased to 67% in HWT-5 min fruit and gradually increased to 50% in HWT-3 min and the control fruit, whereas fruits treated for 1 min had a slight increase (40%). During low-temperature storage, HWT-1 min was given a moderate heat treatment to reduce cold stress, HWT-3 min had no positive effect, and an excessive exposure time—HWT-5 min—triggered cell damage that caused electrolyte leakage that was higher than those in HWT-1 min, HWT-3 min, and the control.

### 3.4. Changes in Lipid Peroxidation

**Figure 6:**
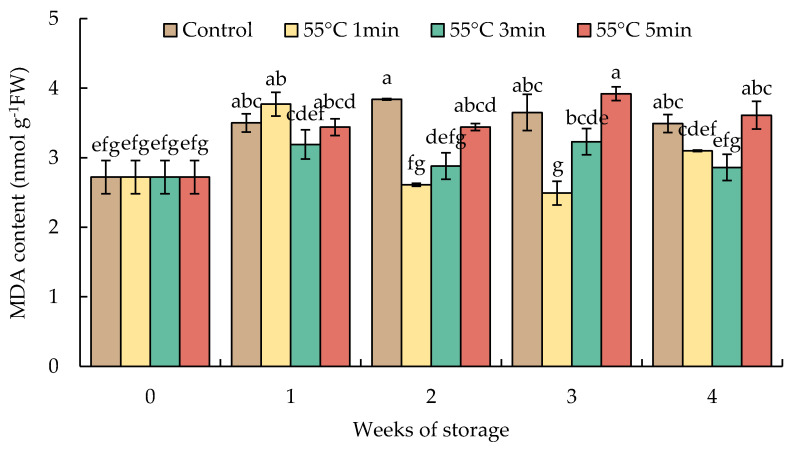
Effect of hot water treatment (55 °C for 1, 3, 5 min) on malondialdehyde (MDA) content in red sweet pepper fruit during storage at 10 °C. Data represent means ± S.E. (n = 3). Different letters on bars are significantly different (*p* < 0.05).

The content of MDA in HWT-1 min fruit markedly increased by week 1, declined thereafter, and remained at a lower level than those of HWT-3 min, HWT-5 min, and the control until the end of storage. There was a slight increase and constant level of MDA in HWT-3 min, while the MDA level in HWT-5 min increased higher than those of shorter treatments, and the content of MDA was the same as that of the control during the storage at 10 °C for 4 weeks (Figure 6). 

### 3.5. Changes in Hydrogen Peroxide Content

**Figure 7:**
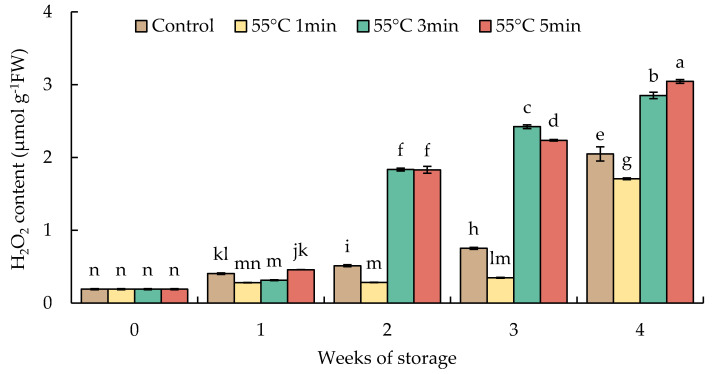
Effect of hot water treatment (55 °C for 1, 3, 5 min) on hydrogen peroxide content in red sweet pepper fruit during storage at 10 °C. Data represent means ± S.E. (n = 3). Different letters on bars are significantly different (*p* < 0.05).

The hydrogen peroxide level increased slightly until week 1 in all fruits. In week 2, the increase was almost 3.6-fold in HWT-3 min and HWT-5 min fruits and continuously increased until the finale of storage; whereas in HWT-1 min fruit, the content of H_2_O_2_ was relatively low and stable from week 1 to week 3 but significantly increased by week 4. HWT-1 min showed a lower H_2_O_2_ content than HWT-3 min, HWT-5 min, and the control (Figure 7). 

### 3.6. Changes in Total Ascorbate and Dehydroascorbate Contents

**Figure 8:**
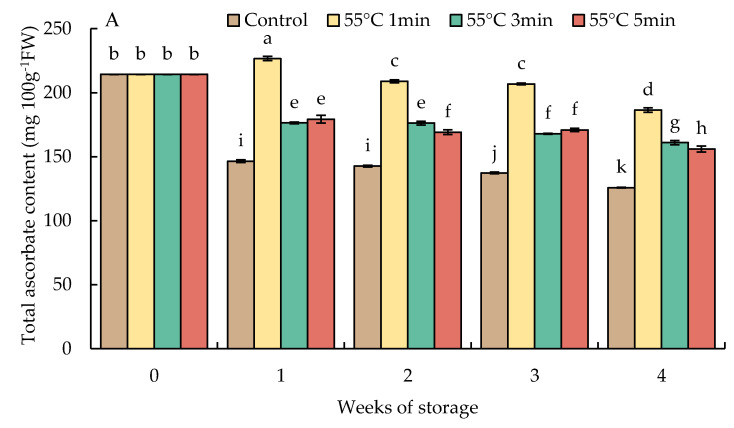

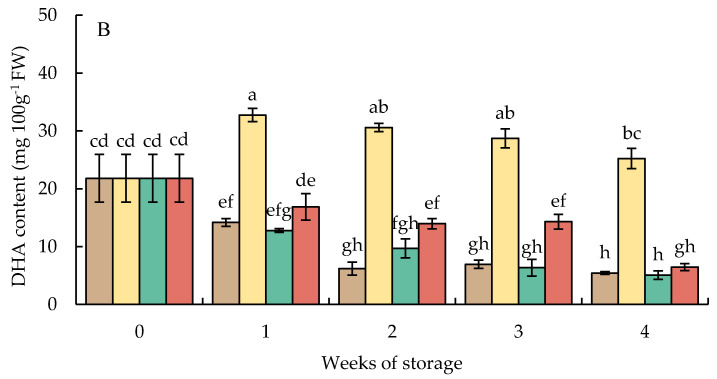
Effect of hot water treatment (55 °C for 1, 3, 5 min) on (**A**) total ascorbate and (**B**) dehydroascorbate (DHA) contents in red sweet pepper fruit during storage at 10 °C. Data represent means ± S.E. (n = 3). Different letters on bars are significantly different (*p* < 0.05).

The level of total AsA in HWT-1 min fruit slightly increased by week 1 but gradually decreased thereafter until the end of storage and was at a higher level than those in HWT-3 min, HWT-5 min, and the control. Prolonged heat treatments (3 and 5 min) showed a lower total AsA level and had a trend similar to that of HWT-1 min (Figure 8A). Heat treatment increased the total AsA and DHA in comparison with the control except at longer exposure times (3 and 5 min), in which excessive treatments induced heat damage in red sweet pepper fruits.

The content of DHA in HWT-1 min fruit increased sharply, by approximately 1.5 times, at 1 week and then gradually declined; the level was higher than that in HWT-3 min, HWT-5 min, and the control regardless of the storage time (Figure 8B).

### 3.7. Changes in Reduced Glutathione and Oxidized Glutathione Contents

**Figure 9:**
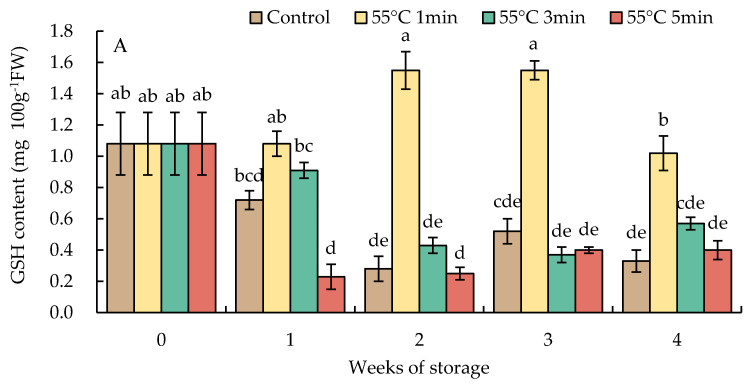

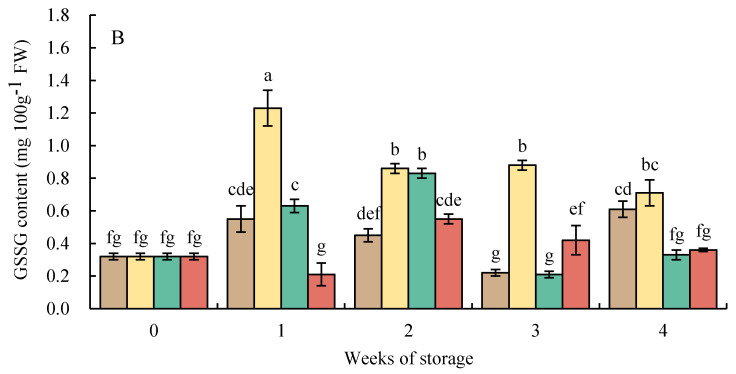
Effect of hot water treatment (55 °C for 1, 3, 5 min) on (**A**) reduced glutathione (GSH) and (**B**) oxidized glutathione (GSSG) contents in red sweet pepper fruit during storage at 10 °C. Data represent means ± S.E. (n = 3). Different letters on bars are significantly different (*p* < 0.05).

The content of GSH in HWT-1 min fruit increased after storage for 1 week and then significantly decreased at week 4; the level was higher than those in HWT-3 min, HWT-5 min, and the control fruit. By contrast, HWT-3 min, HWT-5 min, and control fruits indicated a rapid decline of the GSH content after storage for 1 week and were maintained at a lower level during the storage at 10 °C for 4 weeks (Figure 9A). 

The GSSG level had a tendency similar to that of the level of GSH in HWT-1 min fruit, which sharply increased at week 1 and gradually decreased thereafter, and was higher than those in HWT-3 min, HWT-5 min, and control fruits until the final storage period (Figure 9B). 

### 3.8. Changes in Catalase, Ascorbate Peroxidase, Monodehydroascorbate Reductase, Dehydroascorbate Reductase, and Glutathione Reductase Activities 

Antioxidant enzymes, such as CAT and AsA-GSH cycle-related enzymes (APX, DHAR, MDHAR, and GR), play important roles to maintain cellular homeostasis by scavenging H_2_O_2_ and preventing the accumulation of H_2_O_2_ to toxic levels [14]. 

**Figure 10:**
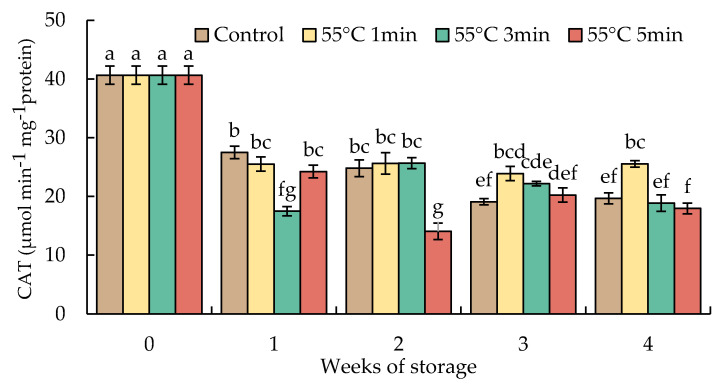
Effect of hot water treatment (55 °C for 1, 3, 5 min) on the activity of catalase (CAT) in red sweet pepper fruit during storage at 10 °C. Data represent means ± S.E. (n = 3). Different letters on bars are significantly different (*p* < 0.05).

The activities of CAT indicated a quick decrease in week 1 and steadily declined until the end of the storage duration in all samples. By week 4, the activities of CAT in HWT-1 min fruit remained at a level higher than those of HWT-3 min, HWT-5 min, and control fruits (Figure 10).

**Figure 11:**
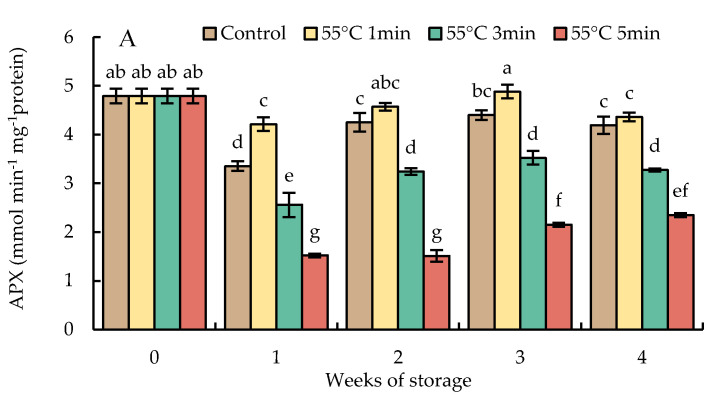

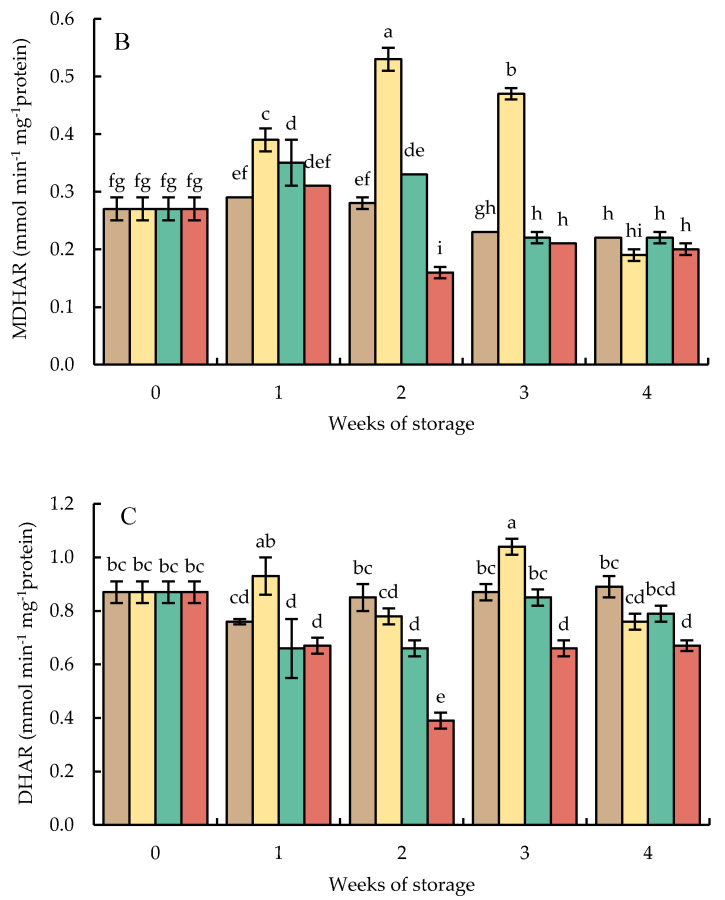

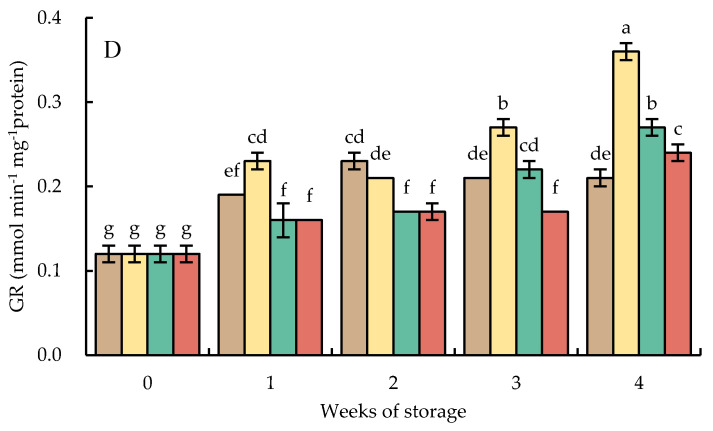
Effect of hot water treatment (55 °C for 1, 3, 5 min) on the activity of (**A**) ascorbate peroxidase (APX), (**B**) monodehydroascobate reductase (MDHAR), (**C**) dehydroascorbate reductase (DHAR), and (**D**) glutathione reductase (GR) in red sweet pepper during storage at 10 °C. Data represent means ± S.E. (n = 3). Different letters on bars are significantly different (*p* < 0.05).

Ascorbate peroxidase activities in HWT-1 min fruit slightly decreased in the first week of storage but increased in weeks 2 and 3, then declined thereafter, and remained higher than those of HWT-3 min, HWT-5 min, and control fruits throughout the storage period. HWT-3 min and HWT-5 min fruits presented a significant decrease in APX activities until the end of storage time, showing a level lower than those of HWT-1 min and control fruits (Figure 11A).

Monodehydroascorbate reductase activities in HWT-1 min fruit sharply increased and approximately doubled after 2 weeks but decreased afterward. The MDHAR activities in HWT-1 min fruit were higher than those in HWT-3 min, HWT-5 min, and control fruits, except in week 4, when all fruits showed similar levels (Figure 11B).

Dehydroascorbate reductase activities in HWT-1 min fruit tended to increase during cold storage for 3 weeks and decreased thereafter. In HWT-1 min fruits, DHAR activities were higher than those of HWT-3 min, HWT-5 min, and control fruits, except in week 4, when all fruits showed similar levels (Figure 11C).

Glutathione reductase activities in HWT-1 min fruit sharply increased during the storage at 10 °C for 4 weeks. The activities of GR in HWT-1 min fruit were higher than those of HWT-3 min, HWT-5 min, and control fruits. There was a gradual increase in GR activities in HWT-3 min and HWT-5 min fruits until the end of storage (Figure 11D).

## 4. Discussion

Low-temperature storage is used extensively to keep the quality and prolong the shelf life of horticultural products after harvest. Nevertheless, because of their sensitivity to cold stress, the utilization of low-temperature storage has some limitations for chilling sensitive horticultural products, such as subtropical and tropical crops [21]. Chilling-induced stress changes the balance between the ROS-forming system and defensive mechanisms, resulting in oxidation-induced CI [10]. 

In the present study, HWT-1 min was effective in alleviating CI and retarded the development of CI symptoms in red sweet pepper fruit as compared to control and the longer treatment times, HWT-3 min and HWT-5 min. CI symptoms presented in the same period of storage (week 2); however, the level of damage differed. Finally, the CI severity in HWT-1 min fruit was slight; however, in the control, HWT-3 min, and HWT-5 min fruit, damage was moderate to severe (Figure 1 and Figure 2). Previous studies also found that hot water immersion modified responses to cold stress, delaying the onset of CI, and thus has been proven to alleviate CI in fresh produce, such as sweet pepper [13,36], mumes [18], plums [37], and cucumbers [16].

Weight loss in untreated red sweet peppers was less than in those treated with hot water, except for HWT-1 min fruit. Hot water immersion increased fruit weight loss in some fresh produce, such as mandarins [38], which lost more weight than the control during cold storage. From this study, short exposure to hot water (HWT-1 min) reduced weight loss (Figure 3), probably as a result of the stimulated recrystallization or molecular orientation of the waxes on the cuticle layer, which plays a significant role in regulating the water loss and preserving fruit firmness [16,39]. However, red sweet pepper fruits exposed to hot water for a longer time (HWT-3 min and HWT-5 min) lost more fresh weight, which might be due to tissue damage (Figure 2). Although hot treatment may be of benefit to hot water-treated horticultural products, excessive heat, such as revelation of products to lethal temperatures or an extended treatment duration, might induce cell damage [40]. Excess heat stress may cause the destruction of cells, the loss of membrane integrity, and the elimination of the wax coating the outer surface of the cuticle, resulting in higher loss of water.

In previous studies, Fallik et al. [41] found serious heat injury in sweet pepper when hot water treatment at 55 °C was applied, and even observed severe fruit damage when treated at 52 °C for 2 min [4,41]. Thus, they [24] suggested 55 ± 1 °C for only 12 s to maintain fruit quality during prolonged storage. In contrast similar with our results (Figure 4), Shehata et al. [42] found that the high-temperature treatment (55 °C for 1 min) provided a good appearance without visible injury and decay in pepper fruits during cold storage. The difference of heat tolerance in fruits depends on species, genotype, stage of fruit maturity, type and severity of heat treatment applied, and the preconditioning treatments before heat treatment [43].

Cell membrane integrity is most affected by CI. In the cell membrane, the transition from the flexible liquid crystalline phase to a solid gel structure phase is caused at cold storage temperatures, which increases the malfunction of cell membranes [44]. When horticultural product is exposed to damaging temperatures below a certain threshold temperature, cell membranes break, resulting in the leakage of ion, metabolites, and intracellular water, which can be traced as electrolyte leakage [21,45]. Electrolyte leakage in fruits treated with hot water immersion differed depending on the exposure time. During storage at 10 °C, brief hot water immersion (HWT-1 min) showed lower electrolyte leakage because mild heat treatment may reduce chilling stress; however, 5 min (HWT-5 min) was an excessive heating time that triggered cell damage and presented the highest leakage (Figure 5). Similarly, treated cucumbers (55 °C, 5 min) had lower electrolyte leakage than untreated fruit, thus hot water mitigated CI by maintaining membrane integrity [16].

Lipid peroxidation is the initial phenomenon caused by CI. Cold stress alters the structure of the plant cell membrane, causing membrane integrity to deteriorate due to lipid peroxidation, which is measured as the MDA level. MDA is the oxidative secondary metabolite of polyunsaturated fatty acid in cell membranes; it is a suitable indicator of oxidative destruction to cell membrane integrity under temperature stress, and its level is a useful indicator of the oxidative stress level [9,11,21]. Our result found that the MDA level in red sweet pepper fruit immersed with HWT-1 min was lower than that with prolonged heat exposure (3 and 5 min) and in the control (Figure 6). The low level of MDA and electrolyte leakage in HWT-1 min fruit reflected the lower degree of CI and the incidence of CI in red sweet peppers during cold storage for 4 weeks. 

Hydrogen peroxide is a strong oxidant, a relatively long-lived molecule, and it is moderately reactive. It is produced by the superoxide dismutase that catalyzes a superoxide radical to H_2_O_2_. Environmental stresses can cause the production of H_2_O_2_; however, excessive H_2_O_2_ can cause oxidative damage in plant cells by disrupting metabolic processes and affecting cell membrane integrity [13,46]. In this study (Figure 7), when comparing different times of heat exposure (55 °C for 1, 3, and 5 min), HWT-1 min fruit had lower H_2_O_2_ contents; this remained constant until week 3 and then rose significantly by week 4. Prolonged heating times (HWT-3 min and HWT-5 min) tended to overheat red sweet peppers, causing higher oxidative stress, which was demonstrated by a higher accumulation of H_2_O_2_ from week 2 until the end of storage (Figure 6). H_2_O_2_ responds to oxidative stress as a signal in the cell compartments that it originates from, leading to an applicable response in the cellular protection system [12]. In low levels, H_2_O_2_ reacts as a signal molecule involved in acclimatory signaling, triggering tolerance to several stresses; in contrast, in high levels, it acts as a providing factor to stress damages and causes cellular destruction [13,47,48]. HWT-1 min could stimulate H_2_O_2_ production to a level that triggered the defense mechanism responses, involving enzymatic and non-enzymatic antioxidative scavenging systems, promoting tolerance of subsequent cold storage. Previous study indicated that temperature stresses reacted to antioxidative scavenging systems in plants. Moderate heat treatment of horticultural produce induces mild oxidative stress, which affects the antioxidant condition and induces tolerance to subsequent stress [18]. The oxidative function of H_2_O_2_ is intimately linked to the redox state in plant tissues. The redox balance is associated with the expression of genes that contribute to stress resistance, stress acclimation, and defense systems [12].

Heat treatment of horticultural crops before storage not only increases their resistance to heat stress but also improves their tolerance to other various stresses [15]. Temperature stress altered homeostasis in plant cell and major processes in its physiological functions, resulting in the accumulation of ROS levels. ROS may potentially play a function in signal transduction processes that activate stress-response pathways and trigger defensive systems [49]. Mild heat treatment causes slight stress in fresh products, stimulating antioxidant responses in both enzymatic and non-enzymatic systems [11]. Non-enzymatic systems include antioxidant substances, such AsA and GSH, whereas antioxidant enzymes involve superoxide dismutase (SOD), CAT, and AsA-GSH cycle-related enzymes (APX, DHAR, MDHAR, and GR) [9,11]. The AsA-GSH cycle consists of an antioxidant and detoxifying system against ROS that has a significant impact on the resistance to chill damage during postharvest storage [50]. Both AsA and GSH are the significant antioxidants in plants that play important roles in stress resistance. High AsA and GSH contents may be responsible for improving performance during postharvest cold storage [51]. AsA is known to be the most powerful antioxidant substance and plays a critical role in maintaining APX activities to detoxify H_2_O_2_ in the AsA-GSH cycle [48]. In the current study (Figure 8A), the content of AsA in HWT-1 min fruit was gradually decreased but maintained at a higher level than those in HWT-3 min, HWT-5 min, and control fruits during the storage at 10 °C. The decreasing trend of DHA contents in HWT-1 min fruit was similar to that of AsA contents (Figure 9B). Prolonged exposure to hot water (3 min and 5 min) decreased the AsA and DHA content. Hot-water dipping has also been observed to increase the AsA level in other fresh products, including tomatoes [9], zucchini [17], and mumes [18]. The elevated redox potential in AsA may correspond with the chill acclimation of hot water-treated fruit. The increased activity of the AsA metabolism system may indicate a key role in lowering H_2_O_2_ levels throughout low-temperature storage [18]. In HWT-1 min fruit, the high level of AsA was reflected in the lower content of H_2_O_2_ during the initial 3 weeks of storage (Figure 7). 

Glutathione has a crucial function in the antioxidative defense mechanism, regenerating AsA from its oxidized form, DHA, throughout the AsA-GSH cycle [48]. In this study (Figure 9), the GSH level in HWT-1 min fruit increased after 1 week of storage and significantly decreased at week 4; throughout the storage period, the content remained higher than in fruits treated differently. Hot water treatment for 1 min increased chilling tolerance by the increase of the GSH production and the GR activities.

The accumulation of ROS induced by various stresses is mitigated by enzymatic protection systems, such as SOD, APX, GPX, and CAT, and non-enzymatic protection systems of low molecular substances, such as AsA, GSH, α-tocopherol, carotenoids, and flavonoids. ROS disturbs many functions of cellular metabolism by injuring nucleic acids, oxidizing proteins, and producing lipid peroxidation [48]. Many processes in plant metabolism may indicate different optimal temperatures, and their related enzymes may be less heat unstable, so they respond differently to the stresses of temperature [18]. CAT is one of the main enzymes that scavenges ROS by decomposing H_2_O_2_ into water and oxygen. The increase in CAT activity is considered to be the adapted characteristic that assists in the disposal of H_2_O_2_. Abiotic stresses either increase or decrease CAT activity, depending on the strength, period, and kind of stress [12]. In this result (Figure 10), hot water treatments were less effective at stimulating CAT activities. CAT activity in red sweet peppers treated with immersion of hot water (55 °C for 1, 3, and 5 min) and untreated fruit tended to decline during cold storage. Similar trends were indicated in mature green mumes [18], which were treated with immersion of hot water (45 °C for 5 min) during low-temperature storage for 4 weeks. Cold stress might cause the downregulation of abundant CAT enzyme protein and the decrease in CAT activity in red sweet peppers during cold storage. 

Ascorbate peroxidase has an important role in the decomposition of H_2_O_2_ and the control of H_2_O_2_ levels in cellular compartments. In the present study (Figure 11A), although HWT-1 min did not significantly increase APX activities, they were higher than those in fruits treated for prolonged times (3 and 5 min) and the control. HWT-1 min might be a modest heat exposure that could induce APX activity to maintain low levels of H_2_O_2_. APX is considered to play a role in the defensive mechanism against CI development during cold storage, and to enhance the tolerance to cold storage, as well as having a more important action in the ROS control or being responsible for regulating ROS signaling [13].

Ascorbate is a powerful antioxidant that can directly detoxify free radicals. It comes in two oxidized forms: MDHA and DHA. In living organisms, the AsA recycling metabolic system is significant for preserving AsA homeostasis against exogenous stimuli. DHAR and MDHAR are two major AsA recycling enzymes and are members of the AsA-GSH cycle [52]. DHAR catalyzes the recycle action of DHA to AsA, utilizing GSH as a hydrogen donor prior to the spontaneous hydrolysis of DHA to irreversibly form 2,3-diketogulonic acid. On the other hand, MDHAR uses reduced ferredoxin or NAD(P)H as an electron donor to recycle MDHA prior to the spontaneous oxidation of MDHA to form DHA [44]. The activity of MDHAR in HWT-1 min fruit highly enhanced and about doubled after 2 weeks of storage but reduced subsequently (Figure 11B). DHAR activity in HWT-1 min fruit slightly increased throughout the first 3 weeks of storage and reduced thereafter (Figure 11C). The levels of both DHAR and MDHAR activities in HWT-1 min fruit were higher than those in HWT-3 min, HWT-5 min, and control fruits. The increase of DHAR and MDHAR activities indicates accumulative responses to cold stress. In a previous study, the higher H_2_O_2_ level produced by heat treatment promoted DHAR and MDHAR activities [13]. Temporally, an increase in gene expression of DHAR and MDHAR was induced in H_2_O_2_ accumulation caused by heat treatment [53]. H_2_O_2_ responds as a molecular-signaling secondary messenger of metabolic control, and regulator of gene expression, increasing responses and activation of protection pathways to various stresses. Thus, the higher activities of DHAR and MDHAR enzymes play a significant role in alleviating CI. 

Glutathione reductase is a key enzyme in the AsA-GSH cycle and catalyzes the conversion of GSSH to GSH by utilization of NADPH as an electron donor. It also plays a crucial role in the defense against ROS by maintaining the reduction state of GSH and the AsA pools [13]. GR activity in HWT-1 min fruit sharply increased and was higher than those in HWT-3 min, HWT-5 min, and control fruits throughout the storage period (Figure 11D). High GR activity induced a larger GSH pool and maintained the AsA-GSH cycle, and consequently enhanced the AsA pool [54]. Therefore, higher levels of GSH content and GR activity imply an influence on chilling tolerance and cold acclimation. The GR has a significant action in resistance systems for chilling stress.

## 5. Conclusions

The findings of our study revealed that pre-treatment with immersion of hot water at 55 °C for 1 min alleviated CI in red sweet pepper fruit during the chilling storage duration, which may be due to the increase of the AsA-GSH cycle by enhanced antioxidant contents of AsA and GSH and the activity of antioxidant-related enzymes. During cold storage, chilling resistance was improved, and the onset of CI was delayed and mitigated in HWT-1 min fruit, as illustrated by low H_2_O_2_ levels, whereas prolonged exposure to hot water (3 and 5 min) caused cellular damage, as showed by increases in weight loss, the CI index, the level of electrolyte leakage, and the MDA level. The non-damaging heat condition of hot water at 55 °C for 1 min can allow cold storage at storage temperatures lower than the optimal temperature. This hot water treatment in the present study is a safe treatment, technologically easier, cheaper, and more feasible for suitable postharvest treatment. 

## Supplementary Materials

The following are available online at https://www.mdpi.com/article/10.3390/foods10123031/s1, Figure S1: Effect of hot water treatment (55 °C for 1, 3, 5 min) on *L**, *a**, *b** color in red sweet pepper fruit during storage at 10 °C.
