# Supplementary material for: Antioxidative Responses to Pre-Storage Hot Water Treatment of Red Sweet Pepper (Capsicum annuum L.) Fruit during Cold Storage
Source: Foods. 2021 Dec 6;10(12):3031. doi: 10.3390/foods10123031 (PMC8701787; doi:10.3390/foods10123031)
Supplement: Supplementary file 1 [file foods-10-03031-s001.zip › Figure S1.pdf]

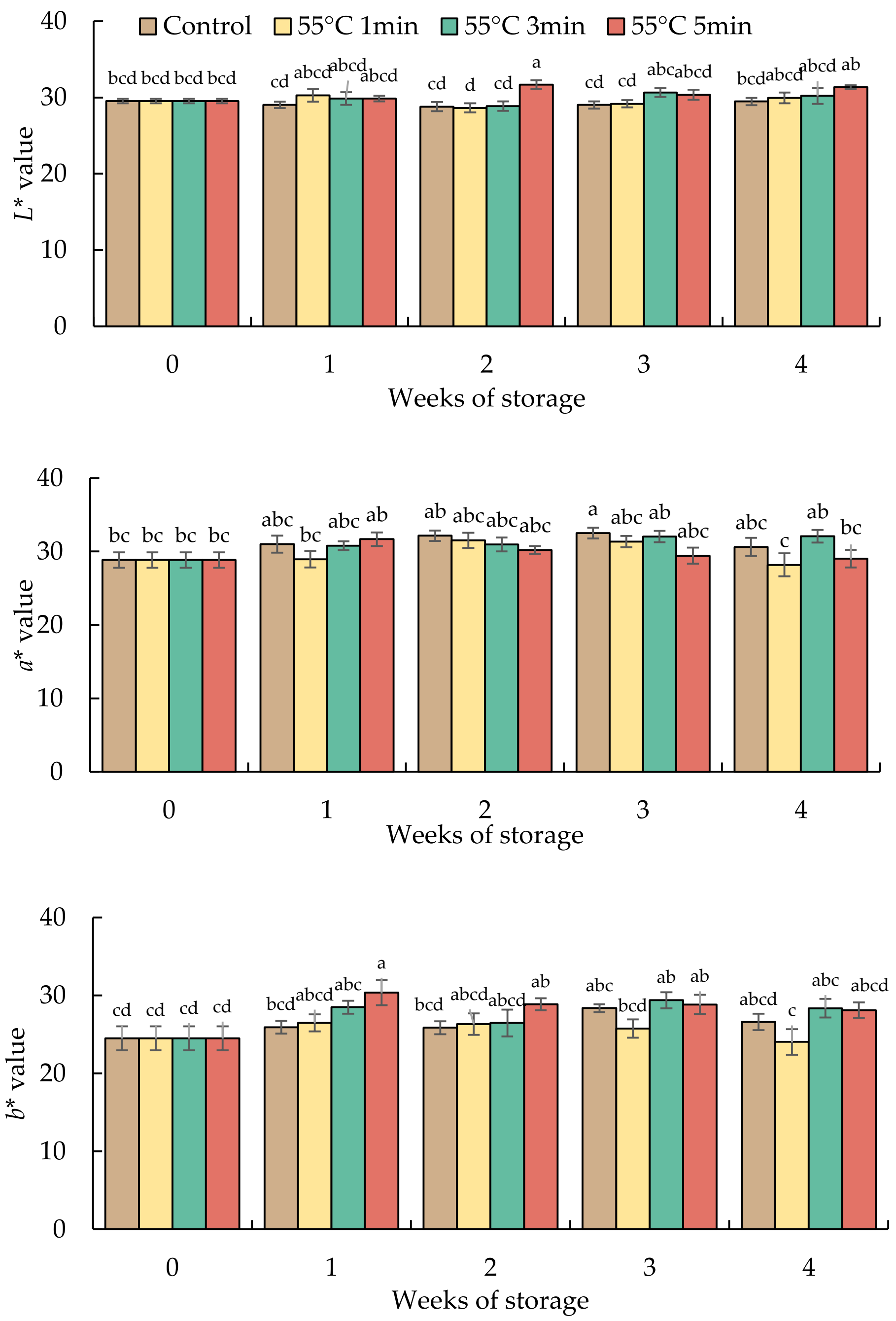

Figure S1. Effect of hot water treatment (55 °C for 1, 3, 5 min) on *L**, *a**, *b** color in red sweet pepper fruit during storage at 10 °C. Data represent means ± S.E. (n = 3). Different letters on bars are significantly different ($P < 0.05$).
